# Individualized osilodrostat treatment for patients with ACTH-dependent Cushing’s syndrome: real-world evidence

**DOI:** 10.3389/fendo.2026.1866631

**Published:** 2026-07-10

**Authors:** Aleksandra Gilis-Januszewska, Aleksandra Gamrat-Żmuda, Mari Minasyan, Martyna Więcławek, Alicja Hubalewska-Dydejczyk

**Affiliations:** 1 Chair and Department of Endocrinology, Jagiellonian University Medical College, Krakow, Poland; 2 Doctoral School of Medical and Health Sciences, Jagiellonian University Medical College, Krakow, Poland

**Keywords:** clinical outcomes, Cushing’s disease, Cushing’s syndrome, dose titration, ectopic Cushing’s syndrome, osilodrostat

## Abstract

**Context:**

Osilodrostat is an 11β-hydroxylase inhibitor that has demonstrated high efficacy in Cushing’s syndrome (CS) in clinical trials; however, real-world data remain limited.

**Objective:**

To evaluate dosing patterns, effectiveness, and safety of osilodrostat in patients with adrenocorticotropic hormone (ACTH)–dependent CS.

**Design:**

This retrospective analysis included 26 adults with ACTH-dependent CS (15 with Cushing’s disease [CD] and 11 with ectopic CS [ECS]) treated with osilodrostat between 2020 and 2025.

**Results:**

Osilodrostat was introduced as first-line therapy in 12 of 26 patients. A titration regimen was used in 21 patients, and a block-and-replace approach in five. A therapeutic effect was achieved in 92% at a median dose of 5 mg/day (4.5 mg/day in CD and 6.5 mg/day in ECS) within 14 days. Morning cortisol normalized in 92% of cases, and urinary free cortisol in 75%. The median clinical score decreased from 9 to 4, and muscle strength increased from 50% to 75% of normal. All 22 patients requiring potassium supplementation showed improvement, with a median time to dose reduction of 10 days. Systolic/diastolic blood pressure decreased from 142/87 to 124/70 mmHg, enabling reduction of antihypertensive therapy in 22 of 23 patients. Among 20 patients with diabetes, 18 reduced the number or dose of antidiabetic medications, including insulin. Adverse events were mild to moderate and included fatigue and nausea (34.6% each), adrenal insufficiency (30.8%), and dizziness (23.1%).

**Conclusions:**

In real-world clinical practice, osilodrostat provided rapid and effective biochemical control and clinical improvement in ACTH-dependent CS, with a manageable safety profile and effective doses in the low-to-moderate range.

## 1 Introduction

Endogenous Cushing’s syndrome (CS) is a severe endocrine disorder caused by excessive cortisol production. The majority of cases (approximately 70–80%) are adrenocorticotropic hormone (ACTH)-dependent (1–3). The predominant cause, accounting for more than 80% of ACTH-dependent cases, is Cushing’s disease (CD), which results from an ACTH-secreting pituitary adenoma. In the remaining cases, hypercortisolism is caused by ectopic ACTH secretion from a non-pituitary neuroendocrine tumor, referred to as ectopic Cushing’s syndrome (ECS) (1–4). Hypercortisolism in CS leads to numerous complications, including diabetes mellitus, hypertension, osteoporosis, neurocognitive impairment, and electrolyte disturbances, making it a potentially life-threatening condition (5–8).

The first-line treatment for endogenous CS is surgical intervention, regardless of etiology (9–11). Surgical remission rates reach approximately 90% in cases of pituitary microadenomas and 50–65% in macroadenomas (9, 12). However, approximately 10–20% of patients experience disease recurrence within the first 5 years after achieving remission. In ECS, management is considerably more complex (13–15). Effective surgical treatment is feasible only when the tumor can be accurately localized and completely resected, with reported success rates of 60–80% in such cases. However, ECS is often diagnosed at an advanced or metastatic stage, in which surgery rarely results in complete remission of hypercortisolism (13, 14). Moreover, in approximately 20% of patients, the source of ectopic ACTH secretion remains unidentified despite repeated biochemical and imaging studies over several years, precluding curative surgical intervention (13, 16). Pharmacological therapy remains the second-line treatment option in these situations (9, 16–19).

Osilodrostat is the most recently introduced adrenostatic agent. It is a potent oral inhibitor of 11β-hydroxylase, the enzyme responsible for the final step in cortisol synthesis.

In 2020, osilodrostat was approved by the U.S. Food and Drug Administration as an effective medical therapy for CD (19). In phase 3 clinical trials involving patients with CD, osilodrostat led to rapid and sustained reduction in cortisol levels, as well as improvement in cardiovascular and metabolic parameters, physical manifestations of hypercortisolism, and quality of life (20–23). In Europe, the drug was approved by the European Medicines Agency in 2020 for the treatment of endogenous CS.

Although randomized controlled trials are essential for establishing the efficacy and safety of new therapies, their design is limited by strict inclusion criteria and controlled conditions. Real-world studies provide valuable complementary evidence by reflecting routine clinical practice and are crucial for optimizing patient management. To date, real-world data have confirmed the effectiveness of osilodrostat in reducing cortisol levels and improving control of comorbidities (24–29). However, aspects such as the rate of dose titration and the determination of the optimal maintenance dose for individual patients remain to be fully characterized in real-world settings.

The aim of this study was to evaluate real-world dosing patterns, clinical efficacy, and adverse effects of osilodrostat therapy in patients with ACTH-dependent CS treated at a tertiary referral center in southern Poland. Additionally, selected cases were presented to illustrate the importance of individualized therapeutic approaches.

## 2 Materials and methods

A retrospective analysis of medical records was conducted in adult patients with ACTH-dependent CS who were treated with osilodrostat at a tertiary referral academic center specializing in pituitary diseases and hypercortisolism between 2020 and 2025. Patients treated with osilodrostat as part of clinical trials conducted at the same center were excluded. All patients were registered in the European Cushing’s Syndrome Registry (ERCUSYN). The final study cohort comprised 26 patients.

The following data were extracted from medical records: demographic information (age and sex) and clinical data, including date of CS diagnosis, etiology of CS, previous treatment modalities (such as prior adrenostatic therapy), the reason for switching to osilodrostat, and disease duration before initiation of therapy. Osilodrostat was administered within the framework of the national compassionate use program (Ratunkowy Dostęp do Technologii Lekowych [RDTL]), which in selected cases resulted in administrative delays in treatment initiation. Additionally, clinical manifestations of hypercortisolism, laboratory parameters, and the degree of control of comorbid conditions were assessed at baseline (before initiation of osilodrostat) and during treatment at the time point corresponding to the best achieved therapeutic response. Because UFC measurements were not consistently available in routine clinical practice owing to limited assay availability and prolonged turnaround times, treatment response was primarily assessed using serum cortisol concentrations. The best achieved therapeutic response was defined as the time point at which a laboratory therapeutic effect (TE) was achieved or, in patients who did not achieve TE, the time point corresponding to the lowest serum cortisol concentration recorded during therapy. A laboratory TE was defined as a decrease in serum cortisol concentration of more than 50% from baseline or the achievement of a morning serum cortisol level below 15 µg/dL.

The clinical presentation, including plethora, striae, fat accumulation in the dorsocervical and supraclavicular regions, proximal muscle atrophy, central obesity, ecchymoses, hirsutism (in women), and amenorrhea (in premenopausal women), was assessed during physical examination using a semi-quantitative Likert scale (0 = absent, 1 = mild, 2 = severe). The maximum possible score was 14 points in men, 16 points in postmenopausal women, and 18 points in premenopausal women. Improvement in physical manifestations of hypercortisolism was defined as any reduction in the score compared with baseline at the time of the best achieved therapeutic response. Muscle strength was assessed using a patient-reported percentage scale ranging from 0% to 100% of perceived normal muscle strength, where 100% represented the patient’s usual pre-disease level of strength. Improvement in muscle strength was defined as an increase of at least 10 percentage points compared with baseline.

Laboratory parameters were also analyzed, including 24-hour urinary free cortisol (UFC), late-night salivary cortisol (LNSC), morning serum cortisol, midnight serum cortisol, and serum potassium levels. Cortisol concentrations in serum, saliva, and urine were measured using validated immunoassays performed in the hospital’s central laboratory according to standard clinical protocols.

Data on osilodrostat use were collected, including initial, maximum, and final or current dose (for ongoing treatment), as well as the time required to reach the maximum dose and the total duration of therapy.

Osilodrostat treatment was individualized according to disease severity and patient characteristics. Patients were typically reassessed every 1–4 weeks, and dose adjustments were guided by biochemical response, primarily serum cortisol concentrations, as well as clinical parameters including blood pressure, glycemic control, muscle strength, and potassium requirements. The total daily dose and its distribution throughout the day were determined by the treating physician based on treatment response and tolerability. The aim of treatment was to achieve biochemical control while minimizing the risk of adrenal insufficiency.

Information on comorbidities and complications of hypercortisolism, including diabetes mellitus, hypertension, and hypokalemia, was also collected. The severity of these conditions and their changes during treatment were evaluated based on the number of medications used (antidiabetic and antihypertensive agents), the degree of glycemic and blood pressure control achieved during therapy, and the need for potassium supplementation.

Time to improvement in glycemic control was defined as the period until a reduction in the dose or number of antidiabetic medications (oral agents or insulin) or normalization of glycated hemoglobin during ongoing treatment. Time to improvement in blood pressure control was defined as the period until a reduction in the dose or number of antihypertensive medications or achievement of normal blood pressure values during therapy. Time to improvement in potassium levels was defined as the period until a reduction in potassium supplementation or a decrease in the spironolactone dose.

Adverse events were also assessed, including adrenal insufficiency, electrolyte disturbances, QT-interval prolongation on electrocardiography, peripheral edema, fatigue, nausea, vomiting, headache, dizziness, and muscle pain.

Descriptive statistics were presented as means with standard deviations, medians with interquartile ranges (25th–75th percentiles), or proportions (%). Statistical analyses were performed using StatsDirect (version 4.0.4).

## 3 Results

### 3.1 Baseline characteristics and previous treatment

Among the 26 identified patients with ACTH-dependent CS, 15 were diagnosed with CD (5 with pituitary macroadenomas and 10 with microadenomas), and 11 had ECS. The etiology of ECS included five cases of small-cell lung carcinoma, one lung adenocarcinoma, one bladder carcinoma, one clear cell carcinoma of the uterus, one pulmonary carcinoid tumor, and two cases of ECS with an unidentified source. The mean age at diagnosis was 56.8 years and was higher in the ECS group (69.2 years) than in patients with CD (47.8 years). Women predominated in the overall cohort (57.7%) and in the CD subgroup (73.3%), whereas men represented the majority of ECS cases (63.6%). The median severity of clinical manifestations of hypercortisolism, as assessed at baseline using the Likert scale, was 9 points. Among features rated as severe (score = 2), dorsocervical and supraclavicular fat accumulation was observed in 42.3% of patients, facial plethora in 38.5%, and striae in 34.6%. All patients presented with proximal muscle weakness, ranging from 15.0% to 60.0% of normal muscle strength.

Hypokalemia was present in 76.1% of patients, with a mean serum potassium concentration of 3.76 mmol/L. The mean morning serum cortisol concentration was 33.8 µg/dL, midnight serum cortisol was 18.9 µg/dL (n = 25), LNSC was 1.47 µg/dL (n = 10), and UFC was 238.7 µg/24 h (n = 16). Before initiation of osilodrostat therapy, diabetes mellitus was present in 76.9% of patients and hypertension in 88.5%. A summary of clinical and biochemical findings, stratified by the etiology of CS, is presented in **Table 1**.

**Table 1:** Baseline demographic, clinical, and biochemical characteristics of patients with ACTH-dependent Cushing’s syndrome treated with osilodrostat.

Parameter	All patients (n = 26)	Cushing’s disease (n = 15)	Ectopic Cushing’s syndrome (n = 11)
Age (years)^a^	56.8 ± 19.8	47.8 ± 21.1	69.2 ± 8.4
Sex, n (%)			
Female	15 (57.7)	11 (73.3)	4 (36.4)
Male	11 (42.3)	4 (26.7)	7 (63.6)
BMI (kg/m^2^)	32.2 ± 7.9	32.5 ± 7.9	31.5 ± 8.4
Muscle strength (% of normal)	50.0 (15.0–60.0)	55.0 (50.0–60.0)	50.0 (15.0–55.0)
Clinical manifestation of CS – Likert scale^b^	9.0 (6.0–13.0)	11.0 (9.0–13.0)	7.0 (4.0–9.0)
Cushing symptoms, n (%)			
Severe buffalo hump	11 (42.3)	9 (60.0)	2 (18.2)
Severe plethora	10 (38.5)	6 (40.0)	4 (36.4)
Severe bruise tendency	6 (23.1)	2 (13.3)	4 (36.4)
Severe stretch marks	9 (34.6)	9 (60.0)	0.0
Hypokalemia, n (%)	20 (76.9)	9 (60.0)	11 (100.0)
Potassium concentration (mmol/L)	3.76 ± 0.81	4.09 ± 0.55	3.31 ± 0.91
Potassium supplementation, n (%)	21 (80.8)	10 (66.7)	11 (100.0)
Urinary free cortisol (μg/24 h)	238.7 (130.7–1968.7) (n = 16)	194.4 (156.9–1007.5) (n = 9)	466.8 (55.0–2272.6) (n = 7)
Late-night salivary cortisol (μg/dL)	1.47 (0.61–3.89) (n = 10)	1.33 (0.32–2.78) (n = 7)	4.15 (0.74–16.00) (n = 3)
Serum cortisol (6 AM) (μg/dL)	33.8 (22.9–45.7)	26.6 (22.7–37.1)	39.2 (33.0–63.4)
Serum cortisol (12 AM) (μg/dL)	18.9 (12.1–30.0) (n = 25)	15.3 (11.2–22.3) (n = 14)	27.2 (14.0–41.0)
ACTH (pg/ml)	154.9 (75.9–185.1)	154.9 (75.9–185.1)	171.0 (75.5–431.5)
Diabetes, n (%)	20 (76.9)	11 (73.3)	9 (81.8)
Hypertension, n (%)	23 (88.5)	12 (80.0)	11 (100.0)
Previous treatment			
Surgery prior to osilodrostat, n (%)	15 (57.7)	14 (93.3)	1 (9.1)
Chemotherapy prior to osilodrostat, n (%)	3 (11.5)	0	3 (27.3)
Previous medical therapy for CS, n (%)			
Metyrapone	11 (42.3)	6 (40.0)	5 (45.5)
Ketoconazole	5 (19.2)	4 (26.7)	1 (9.1)
Pasireotide	2 (7.7)	2 (13.3)	0
Octreotide	1 (3.8)	1 (6.7)	0
Reasons for initiating osilodrostat (if not used as first-line therapy), n (%)	n = 14	n = 8	n = 6
Suboptimal biochemical or clinical effect	9 (64.3)	6 (75.0)	3 (50.0)
Temporary medical therapy pending osilodrostat initiation^c^	4 (28.6)	2 (25.0)	2 (33.3)
Intolerance to previous medication	1 (7.1)	0	1 (16.7)
Time between diagnosis and start of osilodrostat treatment (days)	93.0 (14.2–703.2)	505.0 (33.0–967.0)	16.0 (4.0–75.0)

Data presented as mean ± SD or median (LQ–UQ).

a at diagnosis.

b clinical manifestation of CS—Likert scale: assessment of clinical features of hypercortisolism (plethora, striae, dorsocervical and supraclavicular fat accumulation, proximal muscle atrophy, central obesity, ecchymoses, hirsutism [in women], and amenorrhea [in premenopausal women]) using a semi-quantitative Likert-type scale (0–2), where 0 = absent, 1 = mild, and 2 = severe. The total score ranged from 0 to 14 points in men, 0 to 16 points in postmenopausal women, and 0 to 18 points in premenopausal women.

c refers to temporary medical treatment administered to control hypercortisolism before osilodrostat initiation, pending drug availability via the Polish national compassionate use program (Ratunkowy Dostęp do Technologii Lekowych, RDTL).

Pharmacological treatment in nine patients with CD was initiated due to nonradical pituitary surgery, and in two patients because of disease recurrence after initial surgical remission, with no possibility of reoperation. In four patients, medical therapy was used as bridging treatment prior to surgery. Among patients with ECS, pharmacological therapy was initiated in eight cases due to inoperable disease at diagnosis caused by metastatic spread, in two patients because the source of hypercortisolism could not be identified, and in one patient with a pulmonary carcinoid tumor as bridging therapy before successful surgery. Osilodrostat was used as the first adrenostatic agent in 12 patients. In the remaining patients, prior adrenostatic therapy was switched to osilodrostat in nine cases due to an inadequate biochemical or clinical response, in one case because of drug intolerance, and in four cases, it was administered as short-term bridging therapy while awaiting osilodrostat initiation pending drug availability through RDTL. The mean time from diagnosis to the start of osilodrostat therapy was 93 days (**Table 1**).

### 3.2 Osilodrostat dosage

**Figure 1:**
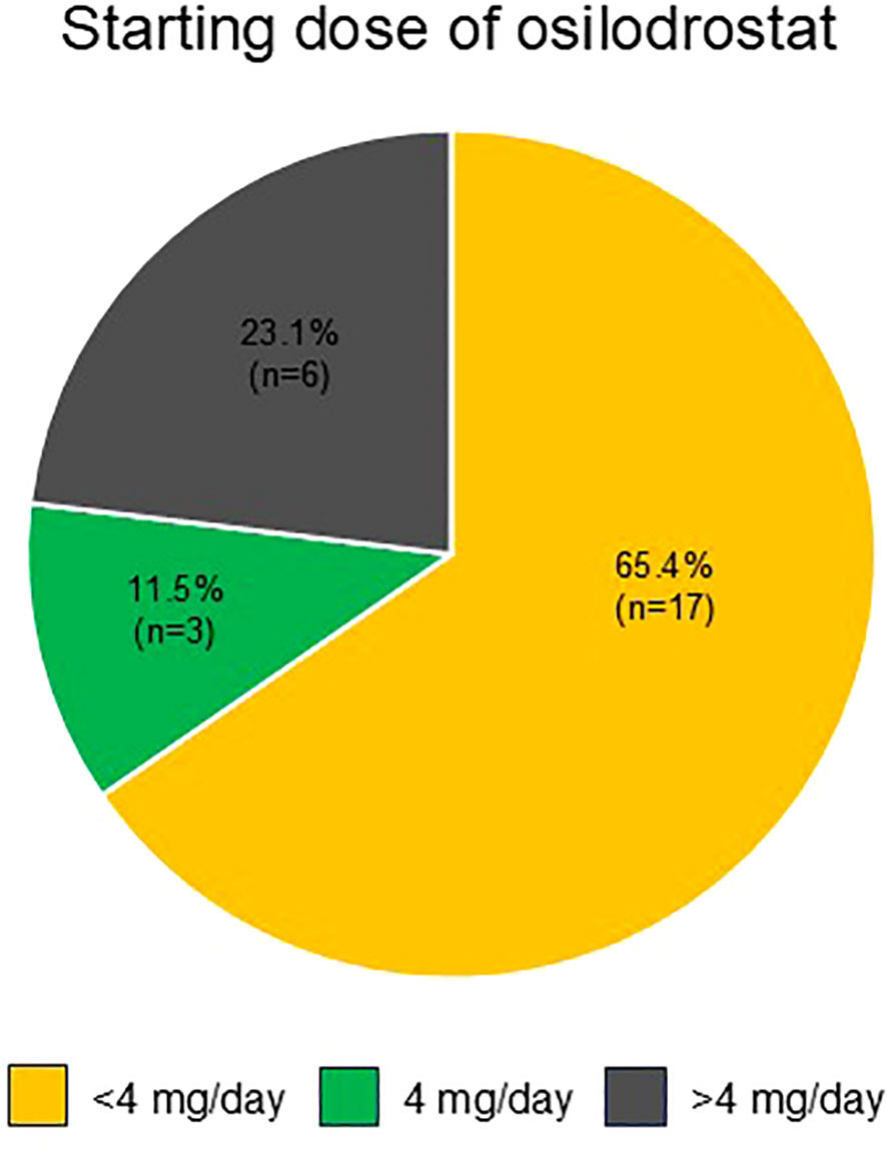
Distribution of initial osilodrostat doses in patients with ACTH-dependent Cushing’s syndrome. The recommended starting dose according to the Summary of Product Characteristics is 4 mg/day.

**Figure 2:**
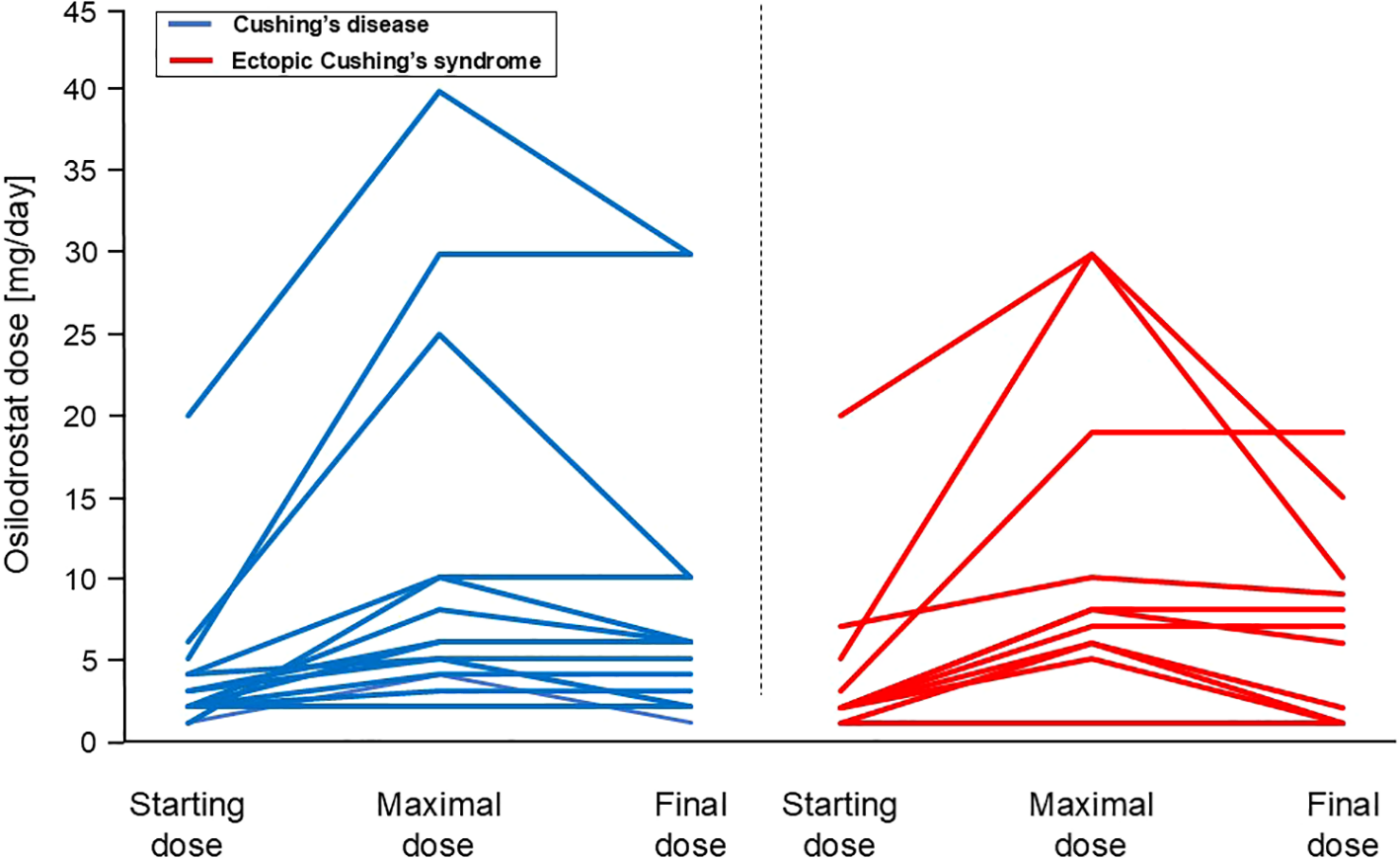
Individual changes in osilodrostat dosing in patients with Cushing’s disease (blue) and ectopic Cushing’s syndrome (red), showing starting, maximal, and final doses during treatment.

Information on osilodrostat dosing is summarized in **Table 2**. In 21 patients, a titration regimen was used, with gradual dose escalation. In four patients, treatment followed a block-and-replace regimen, in which hydrocortisone supplementation was introduced from the start of therapy. In one patient, the block-and-replace approach was initiated after 4 weeks of standard titration. The median starting dose was 2 mg/day, ranging from 1 to 20 mg/day (**Figure 1**). The median maximum dose was 6.5 mg/day in the overall cohort and was higher in patients with ECS (8 mg/day) compared with those with CD (6 mg/day). In eight patients, the maximum dose was ≤4 mg/day. The highest administered dose of 40 mg/day was used in one patient with CD. The median time to reach the maximum dose was 11 days and was shorter in patients with ECS (7 days) than in those with CD (38.5 days) (**Figure 2**). The total duration of therapy ranged from 13 to 1,698 days, with a median of 176.5 days.

**Table 2:** Osilodrostat dosing regimen and treatment characteristics in patients with ACTH-dependent Cushing’s syndrome.

Parameter	All patients (n = 26)	Cushing’s disease (n = 15)	Ectopic Cushing’s syndrome (n = 11)
Osilodrostat dosing regimen, n (%)			
Titration	21 (80.8)	13 (86.7)	8 (72.7)
Block­and­replace^a^	4 (15.4)	2 (13.3)	2 (18.2)
Titration followed by block­and­replace	1 (3.8)	0	1 (9.1)
Starting total daily dose (mg)	2.0 (2.0–4.25)	3.0 (2.0–4.0)	2.0 (2.0–5.0)
Maximum applied dose (mg)	6.5 (5.0–12.25)	6.0 (4.0–30.0)	8.0 (6.0–19.0)
Current/study final dose (mg)	6.0 (2.0–10.0)	6.0 (3.0–10.0)	7.0 (1.0–10.0)
Time to reach maximum dose (days)	11.0 (6.0–75.2)	38.5 (7.7–98.5)	7.0 (3.7–9.5)
Maximum dose treatment duration (days)	22.0 (11.0–87.7)	47.0(19.7–192.2)	12.0 (3.7–23.0)
Duration of therapy (days)	176.5 (49.5–628.7)	189.0 (82.0–973.0)	112.0 (20.0–334.0)
Patients on osilodrostat for ≥6 months to study end, n (%)	13 (50.0)	9 (60.0)	4 (36.4)
Duration of therapy prior to study end in patients with ≥6 months of treatment	585.0 (332.0–1052.0)	930.0 (333.0–1181)	372.0 (331.0–541.2)

Data presented as median (25^th^–75^th^ percentile).

a refers to combined treatment with osilodrostat and hydrocortisone replacement initiated from the start of therapy.

### 3.3 Laboratory effect of osilodrostat treatment

**Figure 3:**
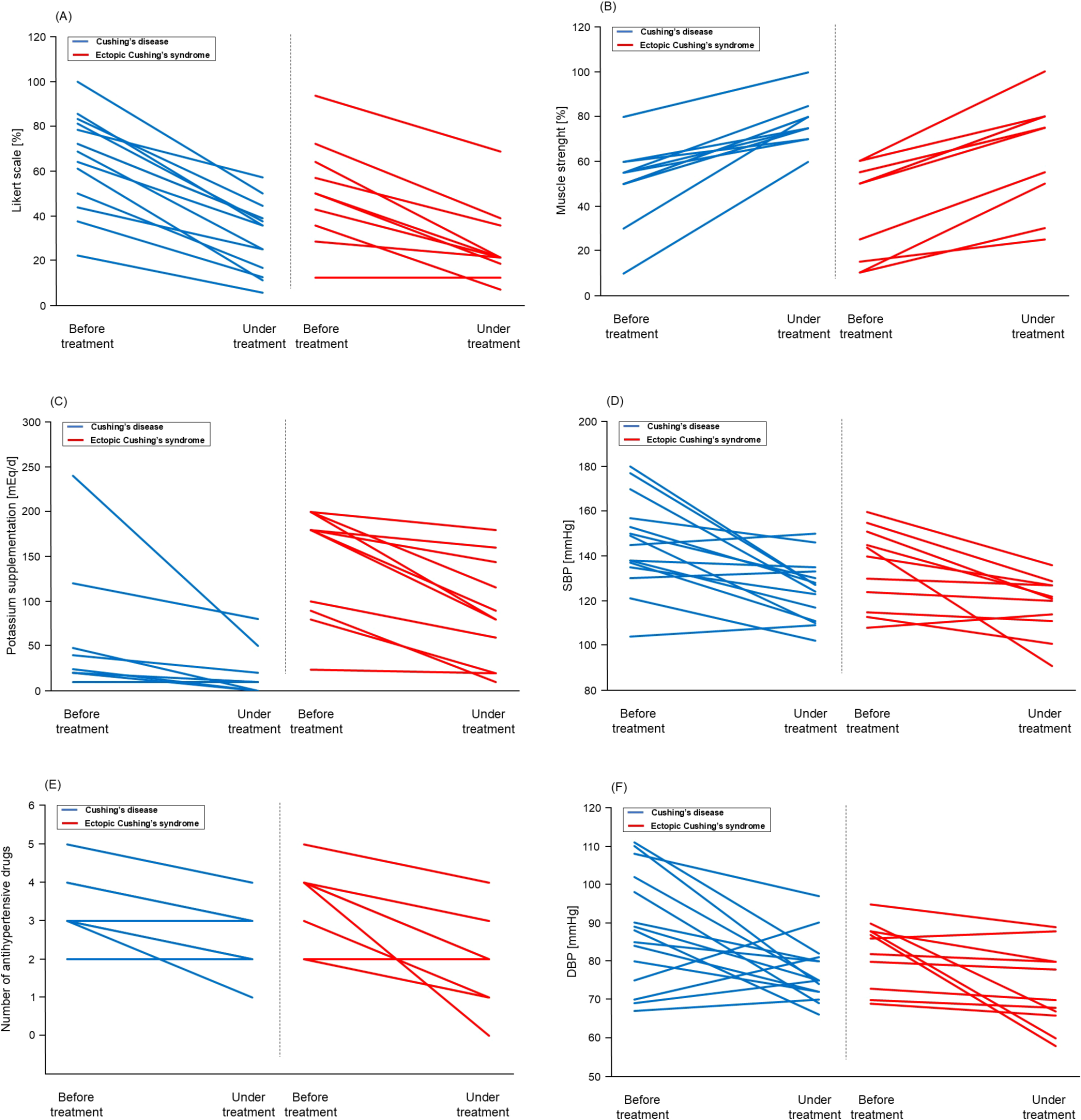
Clinical effects of osilodrostat treatment in patients with ACTH-dependent Cushing’s syndrome. **(A)** change in the Likert scale score1; **(B)** change in muscle strength; **(C)** change in potassium supplementation; **(D)** change in systolic blood pressure (SBP); **(E)** in the number of antihypertensive medications; **(F)** change in diastolic blood pressure (DBP) before and during osilodrostat therapy. Data are presented for individual patients; lines connect pre-treatment and on-treatment values. Patients with ectopic Cushing’s syndrome are shown in red, whereas patients with Cushing’s disease are shown in blue. ^1^Clinical manifestation of CS—Likert scale: assessment of clinical features of hypercortisolism (plethora, striae, dorsocervical and supraclavicular fat accumulation, proximal muscle atrophy, central obesity, ecchymoses, hirsutism [in women], and amenorrhea [in premenopausal women]) using a semi-quantitative Likert-type scale (0–2), where 0 = absent, 1 = mild, and 2 = severe. The total score ranged from 0 to 14 points in men, 0 to 16 points in postmenopausal women, and 0 to 18 points in premenopausal women.

During osilodrostat therapy, a TE was achieved in 24 of 26 patients. Two patients did not achieve TE: one with ECS who was treated for 13 days, and one with CD who was treated for 51 days. The time to achieve TE ranged from 2 to 115 days, with a median of 14 days. The median time to response was 9 days in ECS and 26 days in CD. Therapeutic effect was achieved at osilodrostat doses ranging from 1 to 40 mg/day, with a median of 5 mg/day. The median dose at which TE was achieved was 4.5 mg/day in CD and 6.5 mg in ECS. Among eight patients in whom UFC was assessed and found to be above the upper limit of normal (ULN), normalization was achieved in six. In two patients, UFC levels were within the normal range before treatment. Treatment effects on serum cortisol, salivary cortisol, and UFC are presented in **Figure 3**. During osilodrostat treatment, six patients with CD showed a decrease in ACTH levels (mean reduction of 43%), while five CD patients exhibited an increase (mean rise of 54%). Among patients with ECS, one patient showed an increase in ACTH concentration (by 18%), whereas in eight patients, ACTH levels decreased, with a mean reduction of 54% (**Figure 3**).

### 3.4 Clinical effect of osilodrostat treatment

**Figure 4:**
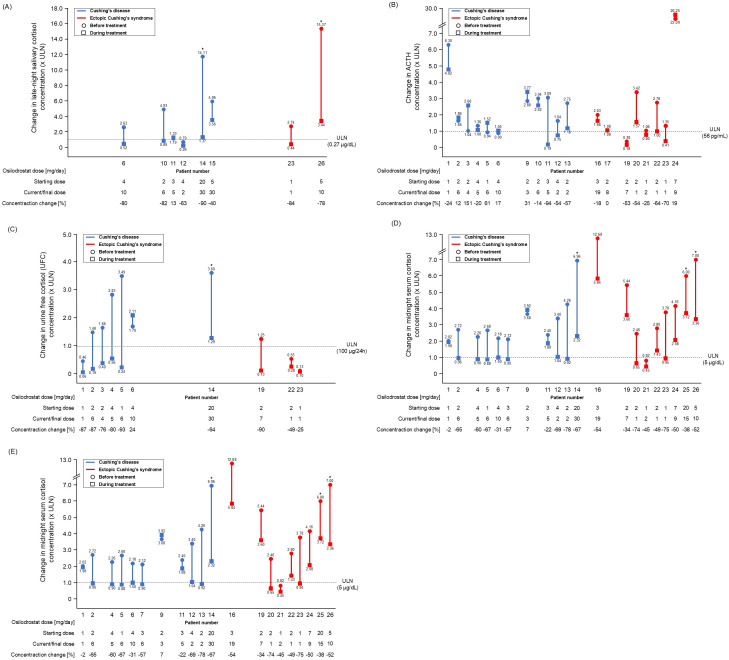
Changes in hormonal parameters during osilodrostat treatment in patients with ACTH-dependent Cushing’s syndrome. **(A)** Morning serum cortisol (6 AM); **(B)** late-night serum cortisol (12 AM); **(C)** late-night salivary cortisol; **(D)** 24-hour urinary free cortisol; and **(E)** plasma ACTH concentrations before and during treatment in patients with Cushing’s disease (blue) and ectopic Cushing’s syndrome (red). Circles represent baseline values, and squares indicate on-treatment values at the time point corresponding to the best achieved therapeutic response. ULN, upper limit of normal.

During osilodrostat treatment, the median severity of clinical manifestations, as assessed by the Likert scale, decreased from 9 to 4 (p < 0.001). The median symptom severity during treatment was higher in patients with CD (5) than in those with ECS (3) (**Figure 4A**). All patients showed improvement in muscle strength (median, 75% of normal strength; median increase, 25 percentage points; p < 0.001). The median muscle strength during treatment was higher in patients with CD (80%) than in those with ECS (75%). The median change in muscle strength was statistically significant in both groups (p = 0.001 in CD and p = 0.003 in ECS) (**Figure 4B**). The time to achieve an improvement of at least 10 percent points ranged from 12 to 85 days, with a median of 35 days. All 22 patients who required potassium supplementation before treatment experienced a reduction in supplementation dose during osilodrostat therapy. The median potassium supplementation dose decreased from 44 to 20 mEq/day (p < 0.001) (**Figure 4C**). The time to any reduction in potassium supplementation ranged from 3 to 98 days, with a median of 10 days; it was shorter in ECS (median, 5 days) than in CD (median, 53 days).

During osilodrostat therapy, the median systolic blood pressure decreased from 142 to 124 mmHg (p < 0.001), and diastolic blood pressure decreased from 87 to 70 mmHg (p < 0.001) (**Figures 4D, F**). A reduction in the number of antihypertensive medications was observed in 22 of 23 patients with hypertension (median, 3 to 2; p < 0.001) (**Figure 4E**). A decrease in the number or dose of antidiabetic medications, including insulin, was observed in 18 of 20 patients with pre-existing diabetes mellitus. The median insulin dose decreased from 18 to 0 units, and the median number of antidiabetic medications from 1 to 0.

### 3.5 Adverse effects of osilodrostat treatment

Adverse events observed during osilodrostat therapy are summarized in **Table 3**. The most commonly reported events were fatigue and nausea (each in 34.6% of patients), followed by adrenal insufficiency (30.8%) and dizziness (23.1%). Headache occurred in 11.5% of patients, while vomiting and myalgia were each reported in 7.7%. Symptoms such as nausea, dizziness, and fatigue partially overlapped with cases of adrenal insufficiency but were also observed in patients without biochemical evidence of adrenal insufficiency. Most adverse events were mild to moderate and resolved after dose adjustment or temporary treatment interruption. Adrenal insufficiency was more frequent in patients with CS (40.0%) than in those with ECS (18.2%) (**Table 3**).

**Table 3:** Adverse events observed during osilodrostat therapy in patients with ACTH-dependent Cushing’s syndrome.

Adverse event	All patients (n = 26)	Cushing’s disease (n = 15)	Ectopic Cushing’s syndrome (n = 11)
Fatigue	9 (34.6)	7 (46.7)	2 (18.2)
Nausea	9 (34.6)	7 (46.7)	2 (18.2)
Adrenal insufficiency	8 (30.8)	6 (40.0)	2 (18.2)
Dizziness	6 (23.1)	6 (40.0)	0
Headache	3 (11.5)	3 (20.0)	0
Vomiting	2 (7.7)	2 (13.3)	0
Myalgia	2 (7.7)	1 (6.7)	1 (9.1)

Data presented as number (%).

### 3.6 Individualized osilodrostat treatment strategy demonstrated in representative clinical cases

Five representative cases are presented to demonstrate the heterogeneity of osilodrostat dosing strategies and clinical responses in patients with CS.

**Figure 5:**
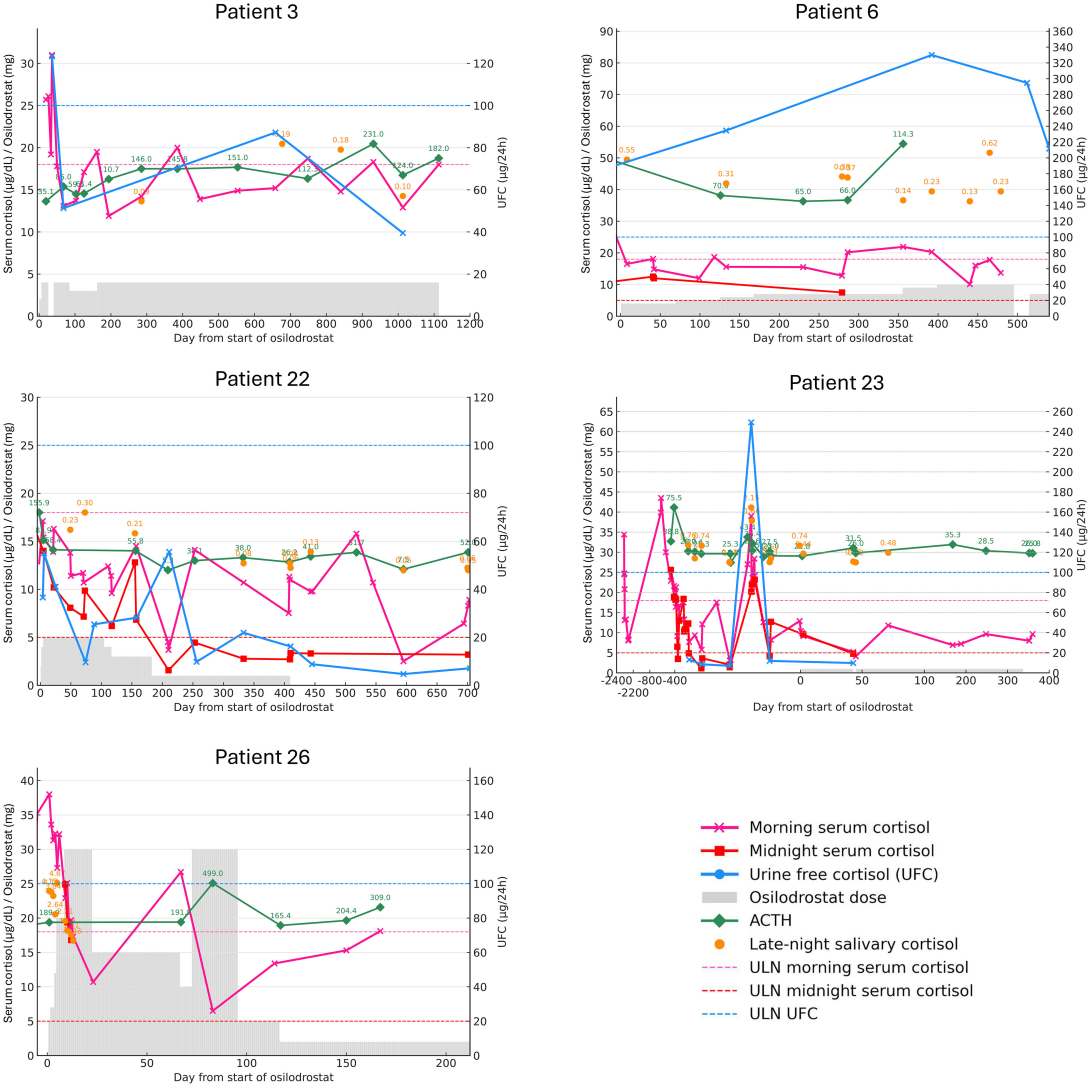
Changes in morning serum cortisol, midnight serum cortisol, urinary free cortisol (UFC), plasma adrenocorticotropic hormone (ACTH), and osilodrostat dose during treatment in patients with Cushing’s syndrome (patients no. 3, 6, 22, 23, and 26). Each panel represents an individual patient.

A 27-year-old woman (no. 3) with CD due to a pituitary microadenoma and severe hepatic impairment (alanine aminotransferase 5×ULN, aspartate aminotransferase 3×ULN), was treated with osilodrostat at a stable dose of 4 mg/day, resulting in biochemical control and normalization of liver enzyme levels. Early in the treatment course, osilodrostat was temporarily discontinued due to coronavirus 2019 (COVID-19); following reintroduction, clinical and biochemical stability was re-established. Treatment has been maintained at the same stable dose for more than 3.5 years, with persistent biochemical remission, sustained clinical stability, and excellent adherence (**Figure 5A**). This case has been previously reported in detail (29).

A 37-year-old man (no. 6) with severe CD caused by a pituitary microadenoma required gradual up-titration of osilodrostat from 4 to 10 mg/day over an 18-month treatment period following nonradical transsphenoidal surgery to maintain adequate biochemical and clinical control (**Figure 5B**).

A 64-year-old man (no. 22) with life-threatening ECS secondary to small-cell lung cancer initially received metyrapone for temporary inhibition of steroidogenesis. After 2 weeks, therapy was switched to osilodrostat. The dose was escalated to 5 mg/day within 7 days, tapered after 3 months, and discontinued after 13 months due to sustained remission of hypercortisolism (**Figure 5C**). Rapid biochemical control of hypercortisolism was accompanied by a marked improvement in general clinical condition, enabling the initiation and continuation of systemic anticancer therapy and ultimately leading to remission. This case was reported in detail previously (30).

A 71-year-old man (no. 23) with cyclic ECS of unknown origin and extreme, unpredictable cortisol fluctuations (5-year history with three documented peaks and three spontaneous troughs) has been maintained on chronic block-and-replace therapy with osilodrostat at a dose of 1 mg/day administered in the afternoon, supplemented with hydrocortisone 10 mg in the morning. This therapeutic strategy enabled stable biochemical and clinical control, allowing effective disease stabilization without episodes of adrenal insufficiency (**Figure 5D**).

A 63-year-old man (no. 26) with ECS secondary to pulmonary adenocarcinoma and life-threatening hypercortisolemia (cortisol concentration up to 38 µg/dL) required rapid escalation of the osilodrostat dose to a maximum of 30 mg/day within 8 days, followed by dose reduction. During the tapering phase, intermittent biochemical relapse of hypercortisolism required transient dose re-escalation to 30 mg/day. Stable biochemical control was ultimately achieved with a maintenance dose of 2 mg/day. Chemotherapy was administered concomitantly with adrenostatic therapy (**Figure 5E**).

## 4 Discussion

In this study of 26 patients with ACTH-dependent CS, normalization of cortisol levels was achieved in 24 patients (92%). This finding confirms the high biochemical efficacy of osilodrostat and is consistent with results from previous prospective and retrospective studies (30–33). In pivotal trials such as LINC-3 and LINC-4 (20–23, 34, 35), treatment efficacy was primarily assessed based on the normalization of 24-hour UFC excretion. In the LINC-3 trial including 137 patients, at least one instance of mean daily UFC normalization was achieved in 96.4% of patients, with 67% maintaining this effect at week 24. In the real-world ILLUSTRATE study involving 42 patients, which included all forms of endogenous CS, UFC normalization was reported in 70% of patients (33). In the current study, UFC data were available for 10 patients; among those with elevated baseline UFC levels, normalization was achieved in 75% of cases.

The limited number of UFC measurements was primarily due to the prolonged turnaround time of the laboratory assay at our center, which reduced its utility for therapeutic decision-making. Similarly, due to limited assay availability and the poor general condition of some patients, salivary cortisol assessment could not be performed in all cases. Given the high rate of morning serum cortisol normalization (92%) observed in our cohort, the potential impact of steroidogenesis precursor interference on immunoassay-based cortisol measurements appears to be minimal. Consequently, serum cortisol measurement remains a reliable tool for evaluating biochemical response, particularly in patients who do not present with elevated UFC levels at baseline (36–40). Moreover, recent findings by Katabami et al. highlight the clinical usefulness of morning serum cortisol as a rapid and reliable marker of both efficacy and safety during osilodrostat therapy (39). The authors demonstrated a strong correlation between morning cortisol and UFC, identifying ≥21.5 µg/dL as a threshold predictive of persistent hypercortisolism, whereas values <5 µg/dL were the most accurate indicator of adrenal insufficiency, even when UFC appeared within the normal range. These observations align with our real-world experience and support the use of serum cortisol as a practical monitoring parameter, particularly when timely dose adjustments are required. Moreover, recent Delphi consensus recommendations highlight that the interpretation of UFC and LNSC is frequently limited by collection errors, biological variability, and delayed availability, prompting experts to emphasize the complementary use of alternative biochemical markers, including morning serum cortisol (40, 41).

In this cohort, the TE—defined as either a ≥50% reduction in serum cortisol or a morning cortisol level <15 µg/dL—was achieved at a median osilodrostat dose of 5 mg/day, with median threshold doses of 4.5 mg/day in patients with CD and 6.5 mg/day in those with ECS. More than 30% of patients required no more than 4 mg/day to achieve biochemical control, corresponding to the recommended starting dose. These findings indicate that effective cortisol suppression can often be achieved with relatively low to moderate doses, although dosing strategies and time to response differ substantially between clinical trials and real-world practice. Previous studies likewise reported effective biochemical control at relatively low-to-moderate doses, although considerable interindividual variability in dose requirements has consistently been observed (33–35, 42). These findings are also broadly consistent with the pooled LINC 2–4 analysis, which demonstrated that the first effective dose most commonly ranged between 4 and 10 mg/day and varied according to baseline cortisol burden, whereas neither the average nor the maximum dose correlated directly with long-term biochemical control (35). In this study, patients with ECS required higher initial doses and achieved biochemical control more rapidly than those with pituitary disease, reflecting the typically more severe hypercortisolism in ectopic forms. However, this was not universal, as the patient requiring the highest maximum dose had CD. Interestingly, patients with CD exhibited more pronounced classical cushingoid features despite lower cortisol concentrations. This may reflect a longer duration of exposure to hypercortisolism, whereas ECS is often characterized by a more rapid clinical course with severe metabolic complications developing before the full spectrum of cushingoid manifestations becomes apparent.

Changes in ACTH concentrations during treatment were heterogeneous. ACTH levels decreased in most patients with ECS and in approximately half of those with CD, although increases were also observed. In patients with ECS, the observed ACTH decline may have been influenced by concomitant treatment of the underlying tumor. Given the retrospective design and limited number of serial ACTH measurements, these findings should be interpreted with caution.

Most patients were treated using a standard titration approach; however, in five cases, initiation or transition to a block-and-replace regimen was necessary. This strategy is particularly beneficial in patients with marked diurnal variability in cortisol secretion or in those at increased risk of transient adrenal insufficiency, such as individuals with cyclic CS (43–45). Recent expert recommendations further propose a severity-driven approach to osilodrostat initiation, advocating more intensive titration or early implementation of a block-and-replace strategy in patients with severe or life-threatening hypercortisolism. This concept is particularly relevant in ectopic ACTH secretion, where rapid biochemical control may be required to stabilize the patient’s clinical condition and enable definitive or oncologic treatment (46). These real-world clinical observations, particularly in patients with paraneoplastic hypercortisolism, are consistent with an individualized, severity-based treatment paradigm.

As illustrated by the presented clinical cases, the dose required to achieve cortisol normalization varied widely and was independent of the underlying etiology of CS. Therefore, the rate of dose escalation should be individualized, taking into account treatment tolerance and the dynamics of the biochemical response. Pharmacodynamic modeling further suggests that the inhibitory effect of osilodrostat on cortisol synthesis is relatively short-acting, supporting the need for individualized dosing schedules, including twice-daily regimens or afternoon administration in selected patients. Previous studies have shown that such a dosing schedule—with highest doses around 4 PM—may help restore the physiological diurnal cortisol rhythm (47). In our clinical practice, dose distribution throughout the day was frequently individualized, with a higher proportion of the daily dose often administered in the afternoon or evening to improve circadian cortisol rhythm, sleep quality, and overall symptom control. As illustrated by patient no. 23, some individuals require only very low doses administered in the afternoon to maintain adequate symptom control.

Moreover, the case of a patient whose treatment was temporarily interrupted due to COVID-19 further highlights the need for personalized therapeutic adjustments and careful evaluation of adrenal insufficiency risk during intercurrent illness or stress (30). It is also noteworthy that elevated liver enzyme activity, commonly observed in patients with active CS, does not constitute a contraindication to osilodrostat therapy (14, 48–50). On the contrary, cortisol reduction may lead to improvement in hepatic function, as observed in patient no. 3.

An essential aspect of treatment efficacy assessment is clinical response. Similar to the LINC-3 trial (22, 51), we used a semi-quantitative clinical scoring system to evaluate symptom severity and observed a comparable reduction of more than 50% relative to baseline values. Furthermore, all 22 patients who required potassium supplementation before treatment showed improvement in electrolyte balance and a reduction in supplementation requirements, representing one of the earliest markers of therapeutic response. Notably, no cases of worsening hypokalemia were observed during therapy, despite this adverse effect being reported in prospective studies as a potential complication of osilodrostat treatment (49, 50, 52). In the retrospective analysis by Castro et al. involving Spanish patients with CD, hypokalemia was likewise not observed as a treatment-related adverse event, which is consistent with these findings (41).

At baseline, all patients presented with reduced muscle strength, which improved markedly during treatment. Although asthenia has been reported as a potential adverse event in clinical trials of osilodrostat and in recent pharmacovigilance analyses (45, 50), our observations suggest that it is rare in clinical practice. This may be related to the rate of dose titration, the speed of cortisol reduction, or individual susceptibility. Therefore, improvement in muscle strength may serve as a clinical marker of effective hypercortisolism control during osilodrostat treatment. Moreover, although previous studies have shown that improvements in proximal muscle function correlate with reductions in UFC (53), muscle strength has not been routinely quantified on a percentage scale as an endpoint in most clinical trials. Our findings indicate that this parameter may represent a valuable and practical indicator of therapeutic efficacy in real-world settings.

As demonstrated in the LINC-3 and LINC-4 studies (34, 35, 51–53), a decrease in cortisol levels translates directly into metabolic improvement. With regard to comorbidities, improvements in glycemic and blood pressure control in this cohort were more pronounced than those observed in prospective trials and other real-world analyses. The proportion of patients requiring reduction of antihypertensive and antidiabetic therapy appeared higher than that reported in previous prospective and real-world studies (33, 51). These discrepancies may reflect a higher proportion of patients with more advanced disease, greater cortisol reduction, and a longer follow-up period at our center, all of which may have contributed to more pronounced clinical and metabolic improvements.

In this cohort, 11 patients had previously been treated with metyrapone and were subsequently switched to osilodrostat due to insufficient biochemical or clinical response, or intolerance to the initial therapy, resulting in improved disease control. This observation is consistent with recent findings from Yokozeki et al., who demonstrated that patients transitioning from metyrapone to osilodrostat may experience additional clinical benefits, including improved blood pressure control and a reduction in androgen-related adverse effects, likely reflecting stronger CYP17A1 inhibition by osilodrostat (54). These findings align with our real-world experience and support the rationale for switching therapy in cases where metyrapone does not provide adequate disease control (54, 55).

This study has several important limitations. Its retrospective design restricts the ability to fully standardize clinical and laboratory data and to establish causal relationships. The relatively small sample size reduces the statistical power of the analysis and limits the robustness of subgroup comparisons (CD vs ECS). Moreover, not all patients had complete biochemical datasets, particularly with respect to UFC and LNSC measurements. However, midnight serum cortisol concentrations were available in a substantial proportion of the cohort and served as an alternative marker of circadian rhythm disruption. In addition, muscle strength was assessed using a patient-reported percentage scale rather than a validated objective measurement tool. Finally, the single-center design may reflect local practice patterns—especially regarding dose titration intensity and monitoring frequency—which may not fully mirror management strategies in other real-world settings.

## 5 Conclusion

Osilodrostat provided rapid and effective biochemical control in ACTH-dependent CS, resulting in substantial clinical and metabolic improvement. Effective doses varied widely, underscoring the need for individualized titration strategies tailored to disease severity and patient tolerance. Overall, osilodrostat demonstrated a favorable safety profile and represents a flexible and reliable therapeutic option in real-world management of hypercortisolism.

## Data Availability

The raw data supporting the conclusions of this article will be made available by the authors, without undue reservation.
